# Gestational weight gain as a risk factor for hypertensive disorders of pregnancy

**DOI:** 10.1016/j.ajog.2013.05.042

**Published:** 2013-10

**Authors:** Corrie Macdonald-Wallis, Kate Tilling, Abigail Fraser, Scott M. Nelson, Debbie A. Lawlor

**Affiliations:** a Medical Research Council Centre for Causal Analyses in Translational Epidemiology, University of Bristol, Bristol, England, UK; b School of Social and Community Medicine, University of Bristol, Bristol, England, UK; c School of Medicine, University of Glasgow, Glasgow, Scotland, UK

**Keywords:** Avon Longitudinal Study of Parents and Children, blood pressure, gestational weight gain, hypertensive disorder of pregnancy, preeclampsia

## Abstract

**Objective:**

Pregnancy interventions to limit gestational weight gain (GWG) have been proposed as a means of preventing hypertensive disorders of pregnancy (HDP); however, it is currently unclear whether GWG has a causal influence on the development of HDP. Thus, we aimed to determine whether GWG in early pregnancy is a risk factor for preeclampsia and gestational hypertension and whether GWG precedes the increases in blood pressure in normotensive women across pregnancy.

**Study Design:**

We examined repeat antenatal clinic measurements of weight and blood pressure (median of 12 and 14 per woman, respectively) of 12,522 women in the Avon Longitudinal Study of Parents and Children.

**Results:**

Greater prepregnancy weight was associated with an increased risk of gestational hypertension and preeclampsia per 10 kg of prepregnancy weight: odds ratio (OR), 1.80; 95% confidence interval (CI), 1.70–1.91 and OR, 1.71; 95% CI, 1.49–1.95, respectively, for women weighing 90 kg or less before pregnancy; OR, 1.24; 95% CI, 1.03–1.49 and OR, 1.61; 95% CI, 1.18–2.19 for women weighing more than 90 kg. Fully adjusted odds ratios for gestational hypertension and preeclampsia per 200 g per week GWG up to 18 weeks were OR, 1.26; 95% CI, 1.16–1.38 and OR, 1.31; 95% CI, 1.07–1.62. In normotensive women, GWG in early pregnancy was associated positively with blood pressure change in midpregnancy and negatively with blood pressure change in late pregnancy. In all gestational periods, GWG was positively associated with concurrent blood pressure change. However, there was no evidence that blood pressure changes in any period were associated with subsequent GWG.

**Conclusion:**

These findings suggest that GWG in early pregnancy may be a potential target for interventions aimed at reducing the risk of HDP.

See Journal Club, page 391

The hypertensive disorders of pregnancy (HDP), including gestational hypertension and preeclampsia, remain a leading cause of maternal and perinatal mortality and morbidity worldwide^1^, ^2^, ^3^, ^4^, ^5^ and can affect up to 10% of pregnancies.^6^ At present, effective treatments are limited, and consequently, there is considerable interest in their prevention. Prepregnancy maternal adiposity is the strongest modifiable risk factor for HDP^7^, ^8^, ^9^, ^10^ and thus provides a potential means of prevention; however, it is increasingly recognized that reducing overweight/obesity in all women of reproductive age is extremely difficult. As a result there has been increased interest in the extent to which gestational weight gain (GWG) may influence HDP and hence provide a potential target for interventions aimed at reducing its risk.^11^

Several studies have shown that HDP are more likely to develop in women who have greater GWG^12^, ^13^, ^14^, ^15^; however, most of these studies have important methodological limitations.^15^ The primary concern is that previous studies have related total GWG across pregnancy to the risk of HDP. This is problematic because women who develop HDP are more likely to experience edema during pregnancy than women who remain normotensive,^16^ and this in turn may result in greater GWG. Therefore, a positive association of total GWG with HDP could represent increased edema in women with HDP, causing greater weight gain, rather than greater GWG influencing increases in blood pressure.^17^ Furthermore, although a recent metaanalysis of interventions designed to limit GWG did show a reduction in HDP,^18^ this could be explained by the treatment of gestational diabetes mellitus that occurred in the 2 largest studies contributing to the metaanalysis.^19^, ^20^

Therefore, clarification of whether greater GWG precedes HDP and therefore could be a potential causal risk factor is urgently needed. The primary aim of this study was to examine whether GWG up to 18 weeks' gestation is associated with the subsequent development of HDP. GWG prior to 18 weeks' gestation, the time of the nadir in blood pressure,^21^ is unlikely to be affected by edema caused by HDP because it occurs before blood pressure begins to increase, and generally clinical edema does not occur until the second half of pregnancy.^22^

To further examine whether GWG influences blood pressure in pregnancy, we also investigated whether greater GWG in different periods of pregnancy precedes greater increases in blood pressure. In this second analysis, we excluded women with HDP to exclude the possibility that the relationships are explained by changes in weight caused by HDP.

## Materials and Methods

The Avon Longitudinal Study of Parents and Children (ALSPAC) is a prospective birth cohort study investigating the health and development of children. The study has been described in full elsewhere^23^ and on the website (Available at: www.bris.ac.uk/alspac. Accessed March 20, 2013). Women with expected delivery dates between April 1, 1991, and Dec. 31, 1992, who were living in a defined area of Avon during their pregnancy were eligible for recruitment. Ethical approval was obtained from the ALSPAC Law and Ethics Committee and from the National Health Service local ethics committee.

A total of 14,541 women were enrolled, 13,678 women had a singleton live birth, and 13,461 of these had data abstracted from obstetric records. We excluded women who reported having a previous diagnosis of hypertension outside pregnancy (n = 445), leaving 13,016 women of whom 12,522 had at least 1 measurement of blood pressure and weight during pregnancy.

### Antenatal blood pressure and weight measurements

All weight and blood pressure measurements (median [interquartile range] per woman: 12 [10–14] and 14 [11–16], respectively) taken routinely as part of antenatal care by midwives or obstetricians were abstracted from obstetric records by 6 trained research midwives. There was no between-midwife variation in the mean values of the data abstracted, and error rates were consistently less than 1% in repeated data entry checks.

Gestational age at delivery was calculated as the difference between the delivery date and the mother's reported last menstrual period date or updated if ultrasound information was available, which led to a reassessment of gestation. At the time of recruitment, first-trimester ultrasound gestational age dating was not routine clinical practice, and the information abstracted from clinical records did not indicate which few women had their gestational age adjusted.

HDP was defined according to International Society for the Study of Hypertension in Pregnancy criteria of systolic blood pressure (SBP) of 140 mm Hg or greater or diastolic blood pressure (DBP) of 90 mmHg or greater, measured on 2 occasions after 20 weeks' gestation for gestational hypertension and the same criteria in conjunction with proteinuria of at least 1+ on dipstick testing for preeclampsia.^24^

Prepregnancy weight was obtained from the predicted weight at 0 weeks' gestation from multilevel models based on the antenatal weight measurements (see the following text). This correlated highly with self-reported prepregnancy weight from a questionnaire administered during pregnancy (r = 0.94). Absolute weight gain was calculated by subtracting the predicted weight at 8 weeks (because there were few measurements prior to this time) from the predicted weight at 40 weeks' gestation, using a multilevel model for weight change across pregnancy including women with term pregnancies (see the following text) and was then categorized according to Institute of Medicine (IOM) recommendations (Appendix; Supplementary Table 1).^17^ Consistent results to those presented here for associations of IOM categories with HDP were found if self-reported prepregnancy weight and the last antenatal weight measurement (in which this was after 37 weeks) were used to define absolute weight gain.

### Maternal characteristics

Maternal age at delivery and offspring sex were abstracted from obstetric records. Maternal height, parity, highest educational qualification, and smoking status were obtained from questionnaires administered during early pregnancy. Prepregnancy body mass index (BMI) was calculated as predicted prepregnancy weight (kilograms)/self-reported height (meters) squared and classified according to the World Health Organization categories (Supplementary Table 1). Smoking status was classed as never for women who did not smoke immediately prior to or during pregnancy, before pregnancy/first trimester for women who smoked only immediately prior to pregnancy or in the first 3 months of gestation, or throughout for women who continued to smoke after the first trimester.

### Statistical analysis

#### IOM categories of GWG and risk of HDP

For women with complete data on IOM categories of GWG and covariates (n = 9596), we used a multinomial regression model to assess the risk of developing gestational hypertension and preeclampsia (with normotensive women as the comparison group) by the IOM category of GWG, adjusting for prepregnancy BMI, maternal age, parity, smoking, education, and sex of offspring.

#### Early pregnancy GWG and risk of HDP

Using all weight measurements up to 18 weeks' gestation for women with at least 1 weight measurement prior to this time (n = 11,760), we developed a multilevel model for the change in weight with gestational age, using a linear slope. The model had 2 levels: measurement occasion (occasion level) within woman (individual level); and 2 individual-level random effects representing weight at 0 weeks (the intercept; referred to as prepregnancy weight) and average GWG per week up to 18 weeks. The individual-level random effects were then used as exposure variables in multinomial logistic regression models.

The outcome variable had 3 categories: normotensive (comparison group), gestational hypertension, and preeclampsia. We adjusted for maternal height, age, parity, smoking, education, and offspring sex, and to examine the exposure of GWG up to 18 weeks, we additionally adjusted for prepregnancy weight. We tested for nonlinearity in these associations using fractional polynomials, and any nonlinear relationships were approximated using linear splines. We also tested for an interaction between prepregnancy BMI category and GWG up to 18 weeks.

#### GWG and changes in blood pressure in normotensive women

We have previously fitted multilevel linear spline models for changes in SBP, DBP,^21^ and weight^25^ with gestational age separately. We combined these models to develop 2 bivariate multilevel linear spline models,^26^ the first including changes in SBP and weight in parallel and the second including changes in DBP and weight in parallel (Supplementary material).

This analysis was restricted to normotensive women who had a term pregnancy (≥37 weeks) (n = 9855). The model with SBP and weight as outcomes had 5 random-effect parameters for SBP: SBP at 8 weeks (baseline) and rate of SBP change at 8-18 weeks (early pregnancy), 18-29 weeks (midpregnancy), 29-36 weeks (late pregnancy), and 36 weeks onward (very late pregnancy) and 4 random-effect parameters for weight: weight at 0 weeks (prepregnancy weight) and rate of GWG at 0-18 weeks (early pregnancy), 18-29 weeks (midpregnancy), and 29 weeks onward (late pregnancy). The model with DBP and weight as outcomes had the equivalent random-effects parameters for DBP as for SBP and the same weight parameters. (Estimates of these parameters are shown in Supplementary Table 2.)

In the second stage, we used the bivariate multilevel models to derive associations of prepregnancy weight and GWG (exposures) with blood pressure change (outcome),^26^ adjusting for maternal height, age, parity, smoking, education, and offspring sex in model 1. In model 2 we additionally adjusted for baseline weight and SBP/DBP and GWG and SBP/DBP changes in periods of pregnancy prior to the exposure period. We then conceptualized GWG as the outcome with changes in blood pressure as a potential risk factor. We therefore derived associations of GWG in each period of gestation (outcome) with SBP/DBP at 8 weeks and SBP/DBP change in previous periods of gestation (exposure).

We adjusted for maternal characteristics in model 1. In model 2 we additionally adjusted for baseline weight and SBP/DBP, GWG, and the SBP/DBP change in all periods prior to the exposure period and GWG in the exposure period.

We completed sensitivity analyses, restricting to women who contributed at least 8 measurements of blood pressure and weight to the bivariate models (n = 6666), and second, restricting to women who contributed no more than 11 measurements (n = 6135). The findings were not meaningfully changed from those in the main analyses. We also performed a sensitivity analysis using an 8 week baseline for weight.

Statistical analyses were carried out using MLwiN version 2.25, STATA version 12.1 and runmlwin^27^ and reffadjust^28^ Stata commands (StataCorp, College Station, TX).

## Results

**Supplementary Figure 1:**
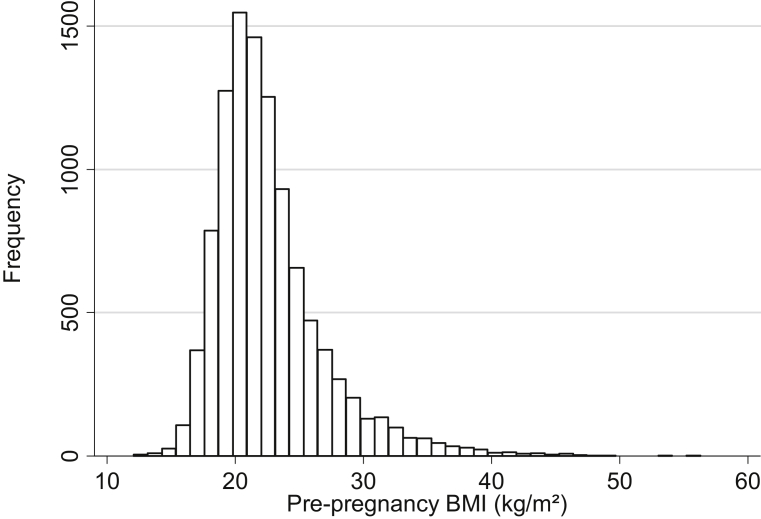
Distribution of prepregnancy BMI (n = 10,411) *BMI*, body mass index. *Macdonald-Wallis. Weight gain and blood pressure in pregnancy. Am J Obstet Gynecol 2013*.

**Supplementary Figure 2:**
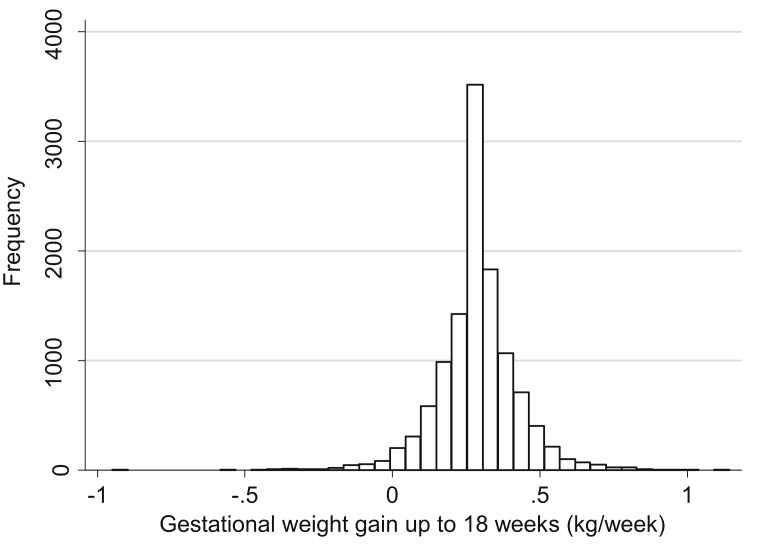
Distribution of GWG up to 18 weeks (n = 11,760) *GWG*, gestational weight gain. *Macdonald-Wallis. Weight gain and blood pressure in pregnancy. Am J Obstet Gynecol 2013*.

The characteristics of women in the full dataset, and subsets with complete data for each analysis, by categories of HDP are shown in Table 1, and distributions of prepregnancy BMI and early-pregnancy GWG are shown in Supplementary Figures 1 and 2, respectively. Each subset had similar characteristics to the full dataset.

**Table 1:** Characteristics of the cohort and subsamples by HDP

Maternal characteristic	Full dataset (total n = 12,522) mean (SD) or %			Complete data for GWG at ≤18 weeks and HDP analysis (n = 9536), mean (SD) or %			Complete data for GWG and BP change analysis (n = 7975) mean (SD) or %
	Normotensive (n = 10,346)	Gestational hypertension (n = 1892)	Preeclampsia (n = 284)	Normotensive (n = 7869)	Gestational hypertension (n = 1466)	Preeclampsia (n = 201)	Normotensive (n = 7975)
Height, cm	(n = 9085)	(n = 1674)	(n = 243)				
	163.81 (6.66)	164.70 (6.95)	162.97 (6.69)	163.88 (6.61)	164.71 (6.96)	162.99 (6.71)	163.94 (6.59)
Age, y	(n = 10,346)	(n = 1892)	(n = 284)				
15-19	5.08	3.96	8.10	3.24	2.52	3.48	3.60
20-24	19.04	21.93	26.41	16.02	19.37	28.36	16.20
25-29	38.49	39.11	34.86	39.69	40.72	35.32	39.44
30-34	27.94	24.68	17.96	30.73	27.08	20.40	30.39
≥35	9.44	10.31	12.68	10.32	10.30	12.44	10.37
Parity	(n = 9610)	(n = 1758)	(n = 255)				
Nulliparous	42.58	57.05	70.98	42.66	57.71	71.64	42.39
Multiparous	57.42	42.95	29.02	57.34	42.29	28.36	57.61
Smoking in pregnancy	(n = 9713)	(n = 1771)	(n = 256)				
Never	65.42	69.85	74.22	68.31	72.24	75.12	68.13
Before pregnancy/first trimester	13.60	14.85	16.41	12.99	14.05	17.91	13.25
Throughout	20.98	15.30	9.38	18.71	13.71	6.97	18.62
Highest qualification	(n = 9253)	(n = 1707)	(n = 242)				
CSE/vocational	30.41	29.23	28.10	28.20	27.49	27.36	28.30
O level	33.99	36.44	36.78	34.63	37.18	36.82	34.51
A level	22.72	21.32	23.55	23.79	22.17	24.38	23.59
Degree	12.88	13.01	11.57	13.38	13.17	11.44	13.61
Offspring sex	(n = 10,346)	(n = 1892)	(n = 284)				
Male	51.29	53.17	51.76	51.14	52.59	53.73	50.51
Female	48.71	46.83	48.24	48.86	47.41	46.27	49.49
Prepregnancy weight, kg^a^	(n = 9855)	(n = 1795)	(n = 209)				
	59.19 (11.07)	67.59 (15.70)	67.76 (17.81)	59.49 (10.65)	67.32 (15.08)	66.38 (16.41)	59.25 (10.83)
Average weight gain up to 18 wks, kg/wk^a^	(n = 9691)	(n = 1804)	(n = 265)				(n = 7186)
	0.290 (0.129)	0.278 (0.148)	0.284 (0.149)	0.291 (0.131)	0.282 (0.150)	0.284 (0.143)	0.292 (0.132)
Total weight gain, kg^a^	(n = 9855)	(n = 1795)	(n = 209)	(n = 7549)	(n = 1399)	(n = 155)	
	13.86 (4.49)	14.96 (5.20)	16.65 (5.59)	13.94 (4.46)	14.98 (5.20)	16.50 (5.77)	13.94 (4.42)

*BP*, blood pressure; *CSE*, certificate of secondary education; *GWG*, gestational weight gain; *HDP*, hypertensive disorders of pregnancy; *SD*, standard deviation.

*Macdonald-Wallis. Weight gain and blood pressure in pregnancy. Am J Obstet Gynecol 2013.*

a Prepregnancy weight is calculated from the predicted weight at 0 weeks' gestation; average weight gain up to 18 weeks is calculated from the predicted rate of weight change between 0 and 18 weeks (for women who had a weight measurement prior to 18 weeks), and total weight gain is calculated as the predicted weight at 40 weeks' gestation minus the predicted weight at 8 weeks' gestation from univariate linear spline random effects models including each woman's antenatal weight measurements.

Nulliparous women, nonsmokers, and women aged over 35 years were more likely to develop HDP (Supplementary Table 3). Gaining more than the IOM recommended weight was associated with an increased risk of gestational hypertension and preeclampsia compared with gaining within the recommended range (Figure; odds ratio [OR], 1.51; 95% confidence interval [CI], 1.32–1.73 and OR, 2.14; 95% CI, 1.46–3.12, respectively). Also, lower than IOM-recommended GWG was associated with a reduced risk of gestational hypertension (OR, 0.75; 95% CI, 0.62–0.91) and preeclampsia (OR, 0.70; 95% CI, 0.38–1.27) compared with GWG within the recommended range (although the confidence interval was wide for preeclampsia and included the null).

**Figure:**
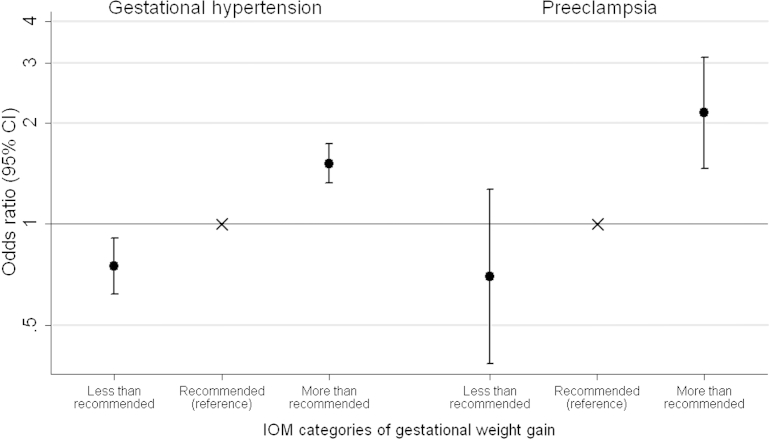
ORs (95% CI) for gestational hypertension and preeclampsia associated with IOM categories of total weight gain (n = 9596)^a^ *CI*, confidence interval; *IOM*, Institute of Medicine; *OR*, odds ratio. ^a^ Adjusted value for maternal prepregnancy body mass index, age, parity, smoking, education, and offspring sex. *Macdonald-Wallis. Weight gain and blood pressure in pregnancy. Am J Obstet Gynecol 2013*.

### Early-pregnancy GWG and risk of HDP

A higher prepregnancy weight was associated with an increased risk of developing gestational hypertension and preeclampsia (Table 2). This association was nonlinear, with evidence of a stronger association between prepregnancy weight and gestational hypertension for women who weighed 90 kg or less than for those who weighed more than 90 kg before pregnancy, although associations with preeclampsia were similar in each group.

**Table 2:** Associations of prepregnancy weight and early-pregnancy GWG with HDP (n = 9536)^a^

Exposure variables^b^	Subgroup	Gestational hypertension		Preeclampsia	
		OR	95% CI	OR	95% CI
Prepregnancy weight (10 kg)	≤90 kg prepregnancy weight	1.80	1.70–1.91	1.71	1.49–1.95
	>90 kg prepregnancy weight	1.24	1.03–1.49	1.61	1.18–2.19
GWG up to 18 weeks (200 g/wk)	All women	1.27	1.16–1.38	1.31	1.07–1.61

*CI*, confidence interval; *GWG*, gestational weight gain; *HDP*, hypertensive disorders of pregnancy; *OR*, odds ratio.

*Macdonald-Wallis. Weight gain and blood pressure in pregnancy. Am J Obstet Gynecol 2013.*

a Both models are adjusted for maternal height (continuous; centered around the mean of 164 cm); age (reference, 25-29 years); parity (reference, nulliparous); smoking (reference, never smoked); education (reference, O level); and offspring sex (reference, male). The model with GWG up to 18 weeks as the exposure is additionally adjusted for prepregnancy weight (2 linear splines: ≤90 kg and >90 kg)

b Prepregnancy weight and GWG up to 18 weeks were obtained using the residuals from a linear multilevel model for weight change up to 18 weeks' gestation. Because of strong evidence for nonlinear associations between prepregnancy weight and these outcomes, results are presented for subgroups of women in whom magnitudes of associations differ.

After adjustment for prepregnancy weight, greater early-pregnancy GWG was independently associated with an increased risk of developing gestational hypertension and preeclampsia (per 200 g/wk increase in GWG up to 18 weeks: OR, 1.26; 95% CI, 1.16–1.38 and OR, 1.31; 95% CI, 1.07–1.62, respectively).

These findings are further illustrated by Table 3, which shows the predicted probabilities of developing gestational hypertension or preeclampsia for different prepregnancy weights and rates of early-pregnancy GWG. This shows clearly that, even among women with healthy prepregnancy weights (eg, 50 or 60 kg), those who gain more weight have important increases in the risk of gestational hypertension and preeclampsia. We found no evidence that associations of GWG up to 18 weeks with the risk of gestational hypertension and preeclampsia differed by prepregnancy BMI category (*P* value for interaction = .18).

**Table 3:** Predicted probabilities of HDP by prepregnancy weight and early-pregnancy GWG (n = 9536)^a^

Prepregnancy weight, kg	GWG up to 18 wks, g/wk	Probability of gestational hypertension, % (SE)	Probability of Preeclampsia, % (SE)
50	200	11.5 (0.9)	1.8 (0.4)
	400	14.1 (1.0)	2.3 (0.5)
	600	17.0 (1.4)	2.9 (0.7)
60	200	19.4 (1.2)	2.9 (0.5)
	400	23.2 (1.4)	3.6 (0.6)
	600	27.3 (2.0)	4.4 (1.0)
70	200	30.7 (1.7)	4.4 (0.8)
	400	35.4 (1.9)	5.3 (1.0)
	600	40.4 (2.6)	6.2 (1.5)
80	200	44.2 (2.3)	6.0 (1.2)
	400	49.3 (2.5)	7.0 (1.5)
	600	54.1 (3.2)	7.9 (2.1)
90	200	57.9 (2.8)	7.5 (1.8)
	400	62.3 (3.0)	8.4 (2.1)
	600	66.1 (3.6)	9.2 (2.8)

*GWG*, gestational weight gain; *HDP*, hypertensive disorders of pregnancy; *SE*, standard error.

*Macdonald-Wallis. Weight gain and blood pressure in pregnancy. Am J Obstet Gynecol 2013.*

a Predicted probabilities are for a woman of average height (164 cm), age (25-29 years), nulliparous, having never smoked during pregnancy, O level educational qualification, and having a male offspring.

### Associations of prepregnancy weight and GWG with subsequent blood pressure change in normotensive women

Analysis of the mean differences in SBP and DBP change for a 200 g/wk increase in GWG in the same and preceding period of gestation are shown in Table 4. For completeness, associations of all possible combinations of prepregnancy weight and GWG in each gestational period with later SBP and DBP changes are also shown in Supplementary Tables 4 and 5, respectively.

**Table 4:** Associations of GWG with subsequent blood pressure change (n = 7975)^a^, ^b^

Exposure Average weight change (200 g/wk)	Outcome Average SBP/DBP change (mm Hg/wk) in the same and subsequent periods of gestation		SBP change		DBP change	
			Mean difference	95% CI	Mean Difference	95% CI
Early pregnancy (0-18 wks)	Same period	Early pregnancy (8-18 wks)	0.06	(0.02–0.10)	0.03	(0.00–0.05)
	Subsequent period	Midpregnancy (18-29 wks)	0.04	(0.00–0.07)	0.03	(0.00–0.05)
Midpregnancy (18-29 wks)	Same period	Midpregnancy (18-29 wks)	0.09	(0.06–0.12)	0.04	(0.01–0.06)
	Subsequent period	Late pregnancy (29-36 wks)	0.00	(–0.06 to 0.06)	0.01	(–0.03 to 0.06)
Late pregnancy (≥29 wks)	Same period (2 parts)	Late pregnancy (29-36 wks)	0.11	(0.06–0.16)	0.08	(0.04–0.12)
		Very late pregnancy (≥36 wks)	0.14	(0.03–0.25)	0.10	(0.01–0.18)

*CI*, confidence interval; *DBP*, diastolic blood pressure; *GWG*, gestational weight gain; *SBP*, systolic blood pressure.

*Macdonald-Wallis. Weight gain and blood pressure in pregnancy. Am J Obstet Gynecol 2013.*

a Adjusted for maternal height, age, parity, smoking, education, and offspring sex; prepregnancy weight; SBP/DBP at baseline; and weight and SBP/DBP change prior to the exposure period (equivalent to model 2 in Supplementary Tables 4 and 5)

b Estimates were obtained from 2 bivariate multilevel spline models: one including all of the women's weight and SBP measurements across pregnancy and the other including all of the women's weight and DBP measurements across pregnancy, restricting to women who had normotensive pregnancies.

In each period of pregnancy, GWG was positively associated with the changes in SBP and DBP that occurred during the same gestational period (Table 4). In addition, an increase in GWG in early pregnancy (up to 18 weeks) was associated with a greater increase in SBP and DBP in the subsequent period of gestation (midpregnancy: 18-29 weeks) (Table 4). However, greater GWG in early pregnancy was also associated with a smaller increase in SBP and DBP in late pregnancy (Supplementary Tables 4 and 5). GWG in midpregnancy (18-29 weeks) was not associated with SBP or DBP change in late pregnancy (29-36 weeks) (Table 4).

### Associations of blood pressure changes with subsequent GWG

To assess whether rises in blood pressure may precede increases in edema and thus weight gain, even in these normotensive women, we also examined associations of changes in SBP and DBP with subsequent GWG (Table 5). All possible associations of baseline SBP and DBP and changes in SBP and DBP with GWG in later periods of gestation are shown in Supplementary Table 6. In fully-adjusted models, there were no associations between SBP or DBP change and GWG in subsequent periods of pregnancy. There was evidence of positive associations between SBP and DBP at baseline and GWG in early pregnancy (Supplementary Table 6). However, when we repeated the bivariate analyses with the baseline set at 8 weeks for weight, neither SBP nor DBP at baseline was associated with GWG in early pregnancy after adjustment for baseline weight (no other findings were changed in this sensitivity analysis).

**Table 5:** Associations of blood pressure changes with subsequent GWG (n = 7975)^a^, ^b^

Exposure Average SBP/DBP change (mm Hg/wk)	Outcome Average weight change (200 g/wk) in the subsequent period of gestation		Mean difference	95% CI
SBP change				
Early pregnancy (8-18 wks)	Subsequent period	Midpregnancy (18-29 wks)	0.06	(–0.06 to 0.18)
Midpregnancy (18-29 wks)	Subsequent period	Late pregnancy (≥29 wks)	–0.01	(–0.17 to 0.15)
DBP change				
Early pregnancy (8-18 wks)	Subsequent period	Midpregnancy (18-29 wks)	0.02	(–0.18 to 0.24)
Midpregnancy (18-29 wks)	Subsequent period	Late pregnancy (≥29 wks)	0.14	(–0.07 to 0.36)

*CI*, confidence interval; *DBP*, diastolic blood pressure; *SBP*, systolic blood pressure.

*Macdonald-Wallis. Weight gain and blood pressure in pregnancy. Am J Obstet Gynecol 2013.*

a Adjusted for maternal height, age, parity, smoking, education, and offspring sex; prepregnancy weight; SBP/DBP at baseline; weight; and SBP/DBP change in all periods of pregnancy prior to the exposure period and weight change in the exposure period (equivalent to model 2 in Supplementary Table 6)

b Estimates were obtained from 2 bivariate multilevel spline models: one including all of the women's weight and SBP measurements across pregnancy and the other including all of the women's weight and DBP measurements across pregnancy, restricting to women who had normotensive pregnancies.

## Comment

In this large cohort, greater prepregnancy weight and GWG in early pregnancy were both independently associated with an increased risk of gestational hypertension and preeclampsia. We also found that, in normotensive women, gaining more weight in early pregnancy was associated with a greater increase in blood pressure in the subsequent period of gestation. In addition, in each period of gestation, GWG was positively associated with the change in blood pressure during the same gestational period. These associations are unlikely to be explained by blood pressure increases leading to increased edema because we found little evidence that blood pressure changes were associated with subsequent GWG in any period of pregnancy in these women.

The review of outcomes associated with GWG by Viswanathan et al^15^ did not find any strong evidence of an association of GWG with risk of HDP. Although the majority of studies reviewed reported a positive association, the review authors noted that the number of studies was limited and most were assessed to be of weak design.^15^ Since the review, 2 studies have reported a positive relationship of excess GWG according to 1990 IOM recommendations with a risk of HDP in 5377 Canadian^29^ and 1043 Latina women,^30^ respectively, and others have reported positive associations between net GWG and HDP risk.^12^, ^13^ However, because IOM criteria combine prepregnancy BMI and GWG over the whole of pregnancy, it is not possible using these criteria to distinguish the impact of prepregnancy BMI from GWG, and none of the studies have addressed whether it is edema associated with HDP that is driving the association.

Our study was limited by the use of routinely collected clinic blood pressure and weight measurements, which is likely to produce random measurement error but unlikely to introduce bias. It also has the advantage that our results could be translated into clinical practice if they are replicated because the measures represent those obtained in clinical practice. Women who develop preeclampsia have reduced plasma volume expansion in early pregnancy compared with women who remain normotensive.^17^, ^31^, ^32^ However, studies have suggested that this is due to increased capillary permeability and a redistribution of plasma to interstitial fluid.^32^, ^33^ We were unable to account for plasma volume expansion in our analyses, but any bias in our findings resulting from the reduction in plasma volume would be expected to attenuate associations toward the null.

The study's strengths are its large size, inclusion of women with a range of BMIs, the high number of repeated weight and blood pressure measurements, and longitudinal modeling of GWG and blood pressure changes in different periods of pregnancy. Because of the repeat measurements of blood pressure and proteinuria, we were able to apply a standard international definition of HDP to all women and not rely on clinical diagnoses.

Outside pregnancy, greater body mass is an established risk factor for high blood pressure,^34^, ^35^ and therefore, the association of greater GWG in early pregnancy with HDP and of GWG throughout pregnancy with blood pressure increases in women without HDP may reflect the effect of greater maternal pregnancy adiposity acquisition on blood pressure.^36^

Also, markers of inflammation, interleukin-6 and C-reactive protein, are increased in obese pregnant women, and this inflammation may contribute to endothelial dysfunction.^37^ Inflammation, measured by C-reactive protein concentrations, has been found in 1 study to mediate the association between prepregnancy BMI and preeclampsia risk.^38^ In our study, we were unable to examine this mechanism. Alternatively, these associations may be partly explained by biological changes related to pregnancy influencing both GWG and blood pressure change or from an underlying predisposition to obesity and cardiovascular risk.^39^

Although our observational data are not able to prove causality, they are a step change from the previous publications and demonstrate temporality by showing that GWG precedes the development of HDP and also a greater increase in blood pressure.

We have also adjusted for a wide range of potential confounders in our analyses. If GWG does have a causal influence on blood pressure change, the potential implications are that limiting GWG in pregnancy may be able to both reduce the incidence of HDP and the severity in those who develop HDP. A recent systematic review and metaanalysis of pregnancy interventions aimed at managing weight gain estimated a 33% reduction in the risk of preeclampsia and a 70% reduction in the risk of gestational hypertension had been achieved through interventions that limited GWG.^18^ However, the 2 largest included studies, which drove the associations, included only women at high risk for gestational diabetes and an intervention aimed at preventing its development. Hence, it is unclear whether the decreased risk of HDP was achieved through the monitoring and management of gestational diabetes because the conditions are strongly linked.^11^

The clinical significance of our findings is indicated by the potential risk reduction for preeclampsia that could be achieved by optimizing GWG. Our results show that the risk of gestational hypertension and preeclampsia is markedly lower in women with lower GWG in all strata of prepregnancy weight (Table 3).

These results combined with evidence from recent trials showing that lifestyle interventions can effectively limit GWG, show the potential to prevent HDP by targeting GWG. Furthermore, the risk reduction that might be achieved by targeting GWG compares favorably with the relative risk reduction of 0.75 (95% CI, 0.66–0.85) observed with antiplatelet agents for the risk of preeclampsia and may be independent of this.^6^ However, any benefits of limiting GWG in terms of reducing HDP risk should be balanced with the potential adverse effects because there is evidence of an increased risk of preterm birth and small-for-gestational-age offspring at the lower end of the GWG distribution.^15^ We were unable to assess the optimum levels of GWG required to achieve this balance in this study.

In summary, we found that greater GWG up to 18 weeks, independently of prepregnancy weight, was associated with a greater risk of developing HDP and in normal pregnancy was associated with a greater midpregnancy rise in blood pressure. GWG in all periods of gestation were positively associated with concurrent rises in blood pressure. This suggests that interventions aimed at limiting GWG from early pregnancy onward may have the potential to reduce the incidence of gestational hypertension and preeclampsia. Our results support establishing randomized trials in nonspecific populations to assess the effectiveness of such interventions in the prevention of these disorders.

## Figures and Tables

**Supplementary Table 1:** IOM-recommended total GWG by prepregnancy BMI category

Prepregnancy BMI category	Recommended absolute weight gain, kg
Underweight (<18.5 kg/m^2^)	12.5-18
Normal weight (18.5-24.9 kg/m^2^)	11.5-16
Overweight (25.0-29.9 kg/m^2^)	7-11.5
Obese (≥30.0 kg/m^2^)	5-9

*BMI*, body mass index; *GWG*, gestational weight gain; *IOM*, Institute of Medicine.

*Macdonald-Wallis. Weight gain and blood pressure in pregnancy. Am J Obstet Gynecol 2013.*

**Supplementary Table 2:** Blood pressure and weight parameters from unadjusted bivariate models (n = 9855)

Blood pressure/weight variable	Mean	Between-individual SD	95% CI reference range
SBP and weight model			
SBP at 8 wks, mm Hg	111.00	7.75	(95.82–126.18)
SBP change, mm Hg/wk			
8-18 wks	–0.177	0.601	(–1.356 to 1.001)
18-29 wks	0.133	0.449	(–0.747 to 1.014)
29-36 wks	0.177	0.625	(–1.048 to 1.401)
≥36 wks	0.967	1.119	(–1.227 to 3.161)
Prepregnancy weight, kg	59.11	11.33	(36.90–81.32)
Weight change, kg/wk			
0-18 wks	0.317	0.228	(–0.129 to 0.763)
18-29 wks	0.525	0.190	(0.154–0.897)
≥29 wks	0.448	0.213	(0.030–0.865)
DBP and weight model			
DBP at 8 wks, mm Hg	65.39	5.48	(54.65–76.13)
DBP change, mm Hg/wk			
8-18 wks	–0.204	0.410	(–1.008 to 0.599)
18-29 wks	0.078	0.326	(–0.560 to 0.717)
29-36 wks	0.313	0.516	(–0.698 to 1.324)
≥36 wks	1.082	1.008	(–0.893 to 3.058)
Prepregnancy weight, kg	59.11	11.33	(36.90–81.32)
Weight change, kg/wk			
0-18 wks	0.317	0.228	(–0.129 to 0.763)
18-29 wks	0.525	0.190	(0.154–0.897)
≥29 wks	0.448	0.213	(0.030–0.865)

*CI*, confidence interval; *DBP*, diastolic blood pressure; *SBP*, systolic blood pressure; *SD*, standard deviation.

*Macdonald-Wallis. Weight gain and blood pressure in pregnancy. Am J Obstet Gynecol 2013.*

**Supplementary Table 3:** Associations of maternal characteristics with HDP

Maternal characteristic	Gestational hypertension		Preeclampsia	
	Unadjusted	Mutually adjusted	Unadjusted	Mutually adjusted
Height (per cm) (n = 11002)	1.02 (1.01–1.03)	1.02 (1.01–1.03)	0.98 (0.96–1.00)	0.98 (0.96–1.00)
Age (n = 12,522), y				
<20	0.77 (0.59**–**0.99)	0.58 (0.40**–**0.82)	1.76 (1.11**–**2.79)	1.09 (0.54**–**2.20)
20-24	1.13 (0.99**–**1.29)	1.13 (0.96**–**1.32)	1.53 (1.13**–**2.08)	1.86 (1.30**–**2.66)
25-29	1	1	1	1
30-34	0.87 (0.77**–**0.99)	0.97 (0.84**–**1.11)	0.71 (0.50**–**1.00)	0.86 (0.58**–**1.27)
≥35	1.07 (0.90**–**1.28)	1.20 (0.98**–**1.46)	1.48 (1.01**–**2.18)	1.90 (1.20**–**3.02)
Parity (n = 11,623)				
Nulliparous	1	1	1	1
Multiparous	0.56 (0.50**–**0.62)	0.53 (0.48**–**0.60)	0.30 (0.23**–**0.40)	0.28 (0.20**–**0.38)
Smoking in pregnancy (n = 11,740)				
Never	1	1	1	1
Before pregnancy/first trimester	1.02 (0.88**–**1.18)	0.91 (0.78**–**1.08)	1.06 (0.76**–**1.49)	0.94 (0.65**–**1.36)
Throughout	0.68 (0.59**–**0.79)	0.67 (0.57**–**0.79)	0.39 (0.26**–**0.60)	0.31 (0.18**–**0.53)
Highest qualification (n = 11,202)				
CSE/vocational	0.90 (0.79**–**1.02)	1.01 (0.88–1.16)	0.85 (0.62**–**1.18)	0.98 (0.69**–**1.39)
O Level	1	1	1	1
A Level	0.88 (0.76**–**1.01)	0.82 (0.70**–**0.95)	0.96 (0.68**–**1.34)	0.90 (0.63**–**1.30)
Degree	0.94 (0.80**–**1.11)	0.81 (0.67**–**0.97)	0.83 (0.54**–**1.28)	0.82 (0.51**–**1.32)
Offspring sex (n = 12,522)				
Male	1	1	1	1
Female	0.93 (0.84**–**1.02)	0.92 (0.82**–**1.03)	0.98 (0.77**–**1.24)	0.94 (0.71**–**1.23)

*CI*, confidence interval; *CSE*, Certificate of Secondary Education; *HDP*, hypertensive disorders of pregnancy; *OR*, odds ratio.

*Macdonald-Wallis. Weight gain and blood pressure in pregnancy. Am J Obstet Gynecol 2013.*

**Supplementary Table 4:** Associations of prepregnancy weight and GWG with subsequent SBP change (n = 7975)^a^

Weight variable		SBP at 8 wks, mm Hg		Average SBP change, mm Hg/wk							
				Early pregnancy (8-18 wks)		Mid-pregnancy (18-29 wks)		Late pregnancy (29-36 wks)		Very late pregnancy (≥36 wks)	
		Mean difference	95% CI	Mean difference	95% CI	Mean difference	95% CI	Mean difference	95% CI	Mean difference	95% CI
Prepregnancy weight (10 kg)											
	Model 1	2.01	(1.70–2.33)	0.04	(0.00–0.08)	–0.07	(–0.09 to –0.04)	0.02	(–0.02 to 0.06)	–0.05	(–0.12 to 0.02)
Average weight change (200 g/wk)											
Early pregnancy (0-18 wks)	Model 1			0.02	(–0.02 to 0.07)	0.05	(0.02–0.08)	–0.09	(–0.14 to –0.04)	0.07	(–0.02 to 0.16)
	Model 2			0.06	(0.02–0.10)	0.04	(0.00–0.07)	–0.10	(–0.15 to –0.05)	0.07	(–0.02 to 0.16)
Midpregnancy (18-29 wks)	Model 1					0.10	(0.07–0.14)	–0.03	(–0.08 to 0.02)	0.07	(–0.02 to 0.16)
	Model 2					0.09	(0.06–0.12)	0.00	(–0.06 to 0.06)	0.04	(–0.06 to 0.14)
Late pregnancy (≥29 wks)	Model 1							0.08	(0.03–0.12)	0.13	(0.05–0.21)
	Model 2							0.11	(0.06–0.16)	0.14	(0.03–0.25)

*CI*, confidence interval; *GWG*, gestational weight gain; *SBP*, systolic blood pressure.

*Macdonald-Wallis. Weight gain and blood pressure in pregnancy. Am J Obstet Gynecol 2013.*

a Model 1 was adjusted for maternal height, age, parity, smoking, education, and offspring sex. Model 2 was adjusted also for prepregnancy weight and SBP at 8 weeks (except for models with prepregnancy weight as the exposure) and GWG and SBP change prior to the exposure period.

**Supplementary Table 5:** Associations of prepregnancy weight and GWG with subsequent DBP change (n = 7975)^a^

Weight variable		DBP at 8 wks, mm Hg		Average DBP change, mm Hg/wk							
				Early pregnancy (8-18 wks)		Midpregnancy (18-29 wks)		Late pregnancy (29-36 wks)		Very late pregnancy (≥36 wks)	
		Mean difference	95% CI	Mean difference	95% CI	Mean difference	95% CI	Mean difference	95% CI	Mean difference	95% CI
Prepregnancy weight (10 kg)											
	Model 1	1.58	(1.35–1.81)	0.01	(–0.02 to 0.04)	–0.05	(–0.07 to –0.03)	0.02	(–0.01 to 0.05)	–0.04	(–0.09 to 0.02)
Average weight change (200 g/wk)											
Early pregnancy (0-18 wks)	Model 1			0.01	(–0.03 to 0.04)	0.04	(0.02–0.06)	–0.04	(–0.08 to –0.01)	0.01	(–0.06 to 0.08)
	Model 2			0.03	(0.00–0.05)	0.03	(0.00–0.05)	–0.05	(–0.08 to –0.01)	0.01	(–0.07 to 0.08)
Midpregnancy (18-29 wks)	Model 1					0.05	(0.02–0.07)	0.00	(–0.04 to 0.03)	0.03	(–0.04 to 0.10)
	Model 2					0.04	(0.01–0.06)	0.01	(–0.03 to 0.06)	0.01	(–0.07 to 0.10)
Late pregnancy (≥29 wks)	Model 1							0.05	(0.02–0.08)	0.08	(0.02–0.15)
	Model 2							0.08	(0.04–0.12)	0.10	(0.01–0.18)

*CI*, confidence interval; *DBP*, diastolic blood pressure; *GWG*, gestational weight gain.

*Macdonald-Wallis. Weight gain and blood pressure in pregnancy. Am J Obstet Gynecol 2013.*

a Model 1 was adjusted for maternal height, age, parity, smoking, education, and offspring sex. Model 2 was also adjusted for prepregnancy weight and DBP at 8 weeks (except for models with prepregnancy weight as the exposure) and GWG and DBP change prior to the exposure period.

**Supplementary Table 6:** Associations of baseline blood pressure and changes in blood pressure with subsequent GWG (n = 7975)^a^

Blood pressure variable		Mean difference in average weight change, 200 g/wk		
		Early pregnancy (0-18 wks)	Mid-pregnancy (18-29 wks)	Late pregnancy (≥29 wks)
SBP at 8 wks (baseline), 5 mm Hg				
	Model 1	–0.05 (–0.08 to –0.01)	–0.04 (–0.07 to –0.02)	–0.02 (–0.05 to 0.02)
	Model 2	0.04 (–0.00 to 0.07)	–0.02 (–0.05 to 0.01)	–0.01 (–0.05 to 0.02)
SBP change, mm Hg/wk				
Early pregnancy (8-18 wks)	Model 1		0.08 (–0.03 to 0.20)	0.05 (–0.08 to 0.18)
	Model 2		0.06 (–0.06 to 0.18)	0.03 (–0.09 to 0.16)
Midpregnancy (18-29 wks)	Model 1			0.21 (0.07–0.37)
	Model 2			–0.01 (–0.17 to 0.15)
DBP at 8 wks (baseline) (5 mmHg)				
	Model 1	–0.07 (–0.13 to –0.02)	–0.06 (–0.11 to –0.02)	–0.05 (–0.10 to –0.00)
	Model 2	0.06 (0.00–0.12)	–0.03 (–0.08 to 0.02)	–0.04 (–0.10 to 0.01)
DBP change, mm Hg/wk				
Early pregnancy (8-18 wks)	Model 1		0.08 (–0.11 to 0.27)	0.12 (–0.09 to 0.34)
	Model 2		0.02 (–0.18 to 0.24)	0.06 (–0.15 to 0.30)
Midpregnancy (18-29 wks)	Model 1			0.29 (0.08–0.50)
	Model 2			0.14 (–0.06 to 0.36)

*CI*, confidence interval; *DBP*, diastolic blood pressure; *GWG*, gestational weight gain; *SBP*, systolic blood pressure.

*Macdonald-Wallis. Weight gain and blood pressure in pregnancy. Am J Obstet Gynecol 2013.*

a Model 1 was adjusted for maternal height, age, parity, smoking during pregnancy, education, and offspring sex. Model 2 was also adjusted for prepregnancy weight and SBP/DBP at 8 weeks, GWG and SBP/DBP in all periods of pregnancy prior to the exposure period, and GWG in the exposure period.

## Appendix

### Supplemental Methods

#### Development of the bivariate linear spline random-effects models

The data were divided into 2 week intervals by gestational age, starting from 4 weeks' gestation, and in cases in which an individual had multiple weight or blood pressure measurements within any 2 week interval, 1 weight and 1 blood pressure measurement was chosen at random from this interval for inclusion in the sample for analysis. This was to prevent individuals with a high number of antenatal visits from having too great an influence on the models. After this process, there remained a median of 10 weight measurements per woman, with a range of 1–17, and a median of 10 blood pressure measurements per woman, with a range of 1–17.

Univariate linear spline models have previously been fitted to systolic blood pressure (SBP), diastolic blood pressure (DBP)^1^ and weight.^2^ Separate models were fitted with SBP, DBP, and weight as the outcome variables (Y), and for each model, gestational age in weeks was used as the exposure variable (X). The models had 2 levels: antenatal visit and individual because there were multiple antenatal visits per woman. The knot points (indicating changes in slope) for these models were determined by initially fitting fractional polynomial curves^3^ to each of SBP, DBP, and weight to describe the shape of the average patterns of change with gestational age. Models with 2 or 3 knots were considered and the final positioning and number of knots was selected as that which optimally fulfilled the criteria of a high model log likelihood, a close fit to the fractional polynomial curve, and good fit of the model predicted values to observed values over the whole course of pregnancy. The best-fitting models for both SBP and DBP had 3 knots at 18, 30, and 36 weeks' gestation, and the model for weight had 2 knots at 18 and 28 weeks' gestation. Each contained individual-level random effects for the intercept and each of the splines.

Because the second knot point for SBP and DBP (at 30 weeks) was in a similar location to the second knot for weight (28 weeks), for the bivariate spline models, we shifted this knot point 1 week earlier for SBP and DBP and 1 week later for weight and used knots at 18, 29, and 36 weeks for SBP and DBP and knots at 18 and 29 weeks for weight. This was for simplicity and interpretability so that the first 2 periods of blood pressure change and gestational weight gain (GWG) had the same endpoints and did not substantially alter the fit of the models.

Few measurements were available prior to 8 weeks so the baseline was set at 8 weeks' gestation for SBP and DBP because blood pressure begins to decrease early in pregnancy but was set at 0 weeks for weight because weight changes little in very early pregnancy. The coefficients from these unadjusted bivariate models model are shown in Supplementary Table 2.

We then added the maternal characteristics of height (continuous), age (<20, 20-24, 25-29, 30-34, ≥35 years), parity (nulliparous, multiparous), smoking (never, before pregnancy/first trimester/throughout pregnancy), education (certificate of secondary education [CSE]/vocational, O level, A level, degree), and offspring sex (male/female) into each of these models as main effects and interactions with each of the splines, with separate effects for SBP/DBP and weight.

#### Derivation of associations of changes in weight with changes in blood pressure from the bivariate multilevel spline models

We derived associations of the weight and GWG parameters with baseline SBP/DBP and SBP/DBP change parameters using the variance-covariance matrix of random effects from each of the models using formulae described by Macdonald-Wallis et al^4^ implemented using the REFFADJUST package in STATA (StataCorp, College Station, TX).^5^

Adjustment for previous GWG and SBP/DBP changes was also made using the variance-covariance matrix of random effects. Because the variances and covariances of the random effects were estimated with uncertainty in the bivariate models, for both of the models, we generated 10,000 realizations of each of the variances and covariances of the random effects from a multivariate normal distribution using the means, variances, and covariances of these estimates calculated by the bivariate random-effects models. We then averaged regression coefficients over the 10,000 realizations and formed 95% confidence intervals for the regression coefficients using the 2.5th and 97.5th percentiles of the distributions of these regression coefficients over the 10,000 realizations to fully incorporate the uncertainty in the estimation procedure.^4^
